# Antarctic sea ice region as a source of biogenic organic nitrogen in aerosols

**DOI:** 10.1038/s41598-017-06188-x

**Published:** 2017-07-20

**Authors:** Manuel Dall’Osto, Jurgita Ovadnevaite, Marco Paglione, David C. S. Beddows, Darius Ceburnis, Charlotte Cree, Pau Cortés, Marina Zamanillo, Sdena O. Nunes, Gonzalo L. Pérez, Eva Ortega-Retuerta, Mikhail Emelianov, Dolors Vaqué, Cèlia Marrasé, Marta Estrada, M. Montserrat Sala, Montserrat Vidal, Mark F. Fitzsimons, Rachael Beale, Ruth Airs, Matteo Rinaldi, Stefano Decesari, Maria Cristina Facchini, Roy M. Harrison, Colin O’Dowd, Rafel Simó

**Affiliations:** 1 Institut de Ciències del Mar, CSIC, Pg. Marítim de la Barceloneta 37-49, 08003 Barcelona, Catalonia Spain; 2 School of Physics and Centre for Climate and Air Pollution Studies, Ryan Institute, National University of Ireland Galway, University Road, Galway, Ireland; 3 Institute of Atmospheric Sciences and Climate, National Research Council, Bologna, 40129 Italy; 4 National Centre for Atmospheric Science, The School of Geography, Earth and Environmental Sciences, The University of Birmigham, Edgbaston, Birmingham, B15 2TT United Kingdom; 5 Biogeochemistry Research Centre, University of Plymouth, Drake Circus, Plymouth, PL4 8AA UK; 6 Instituto INIBIOMA, CRUB Comahue, CONICET, Quintral 1250, 8400S.C. de Bariloche, Rio Negro, Argentina; 7 Department of Evolutionary Biology, Ecology and Environmental Sciences, Universitat de Barcelona, Av. Diagonal 643, 08028 Barcelona, Catalonia Spain; 8 Plymouth Marine Laboratory, Prospect Place, Plymouth, PL1 3DH UK; 9 Department of Environmental Sciences / Center of Excellence in Environmental Studies, King Abdulaziz University, PO Box 80203, Jeddah, 21589 Saudi Arabia

## Abstract

Climate warming affects the development and distribution of sea ice, but at present the evidence of polar ecosystem feedbacks on climate through changes in the atmosphere is sparse. By means of synergistic atmospheric and oceanic measurements in the Southern Ocean near Antarctica, we present evidence that the microbiota of sea ice and sea ice-influenced ocean are a previously unknown significant source of atmospheric organic nitrogen, including low molecular weight alkyl-amines. Given the keystone role of nitrogen compounds in aerosol formation, growth and neutralization, our findings call for greater chemical and source diversity in the modelling efforts linking the marine ecosystem to aerosol-mediated climate effects in the Southern Ocean.

## Introduction

Antarctic sea ice covers between 1% (summer) and 5% (winter) of the global ocean, and influences an even larger area, the sea ice region. It is a vast biome composed of multiple habitats such as the upper and lower ice surfaces, snow cover, brine channels, melt ponds, ice openings and ice floes of all sizes, and the surrounding sea water, all hosting rich microbial communities^1^. Climate warming-derived changes in sea ice extent^2^, thickness and texture are expected to exert feedback effects on climate through changes in surface albedo and emission of climate-active substances, such as those forming cloud-seeding aerosols^3, 4^. An accurate projection of climate dynamics in the Southern Ocean, therefore, requires a sound assessment of aerosol precursor emissions from the sea ice region ecosystem. Here we report results of a study conducted during a research cruise across the Subantarctic and Antarctic waters of the Atlantic sector of the Southern Ocean in summer 2015, where low troposphere aerosols were characterised, source apportioned and compared with concurrent measurements in sea water and sea ice.

## Results and Discussion

**Figure 1:**
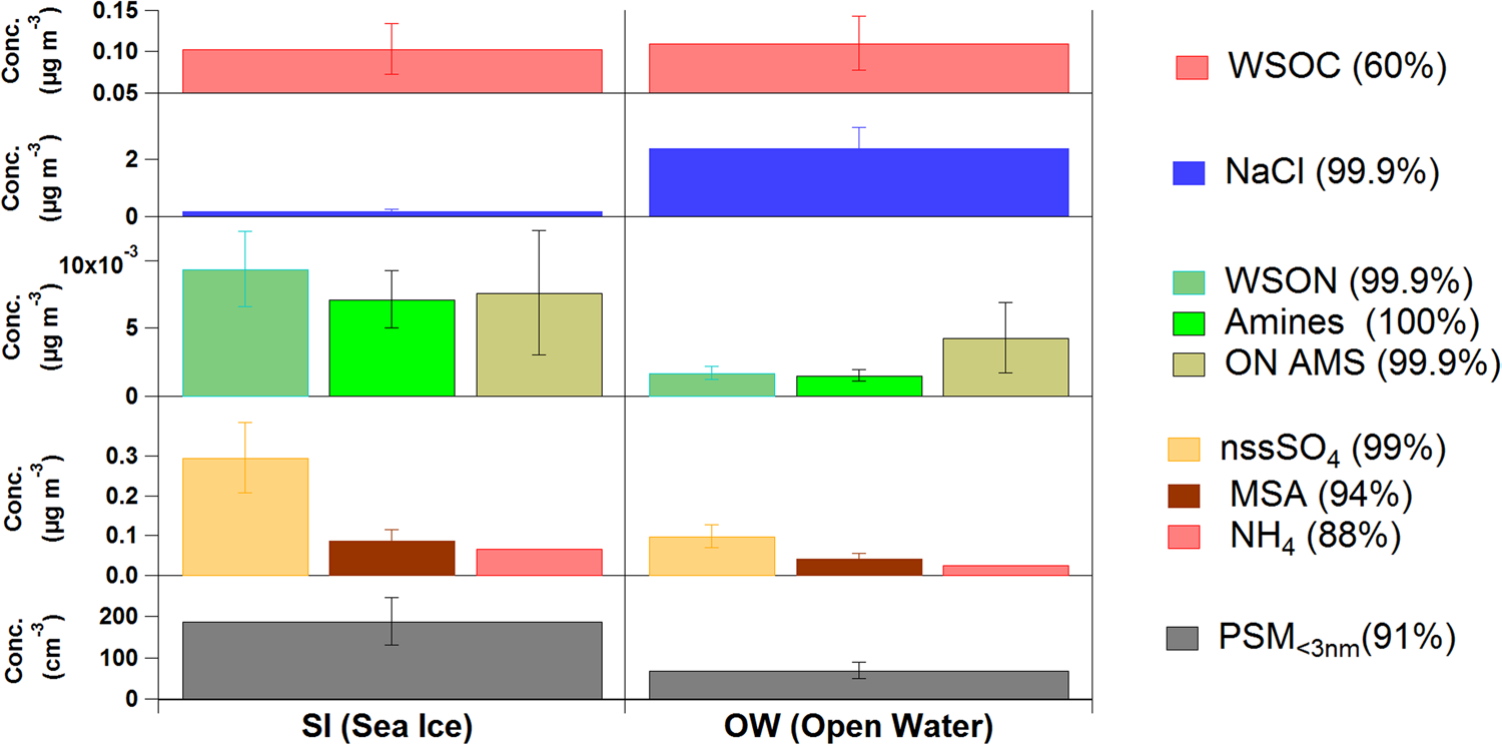
Characteristics of collected aerosols according to their origin. Average and standard deviations are given for aerosol chemical composition and ultrafine particle concentrations in air masses with (left, SI, n = 3) and without (right, OW, n = 3) sea ice influence. WSOC, NaCl, WSON, amine (sum of alkyl-amines), nssSO_4_, MSA and NH_4_ (ammonium) are given as mass collected on filters; ON AMS is organic nitrogen-containing m/z fragments measured continuously *in situ*; N is number concentration of particles in the size range 1–3 nm. The % probability that SI and OW averages are different is given in parenthesis.

Air mass trajectories were used to define “open water” (OW) and “sea-ice” (SI) source regions according to the characteristics of the area overflown (Supplementary Figure 1 and Table 1). A thorough examination of air mass trajectory heights showed that > 80% of the time during the 5 days prior to sampling, the collected air had travelled within the marine boundary layer, with very little intrusion from the free troposphere, and there was no significant differences between SI and OW air masses in this respect (Figures S2, S3 and Table S2). Filter-collected aerosols arriving from OW regions had ten-fold more sea salt and similar amounts of water soluble organic carbon (WSOC) than aerosols originated in the SI region (Fig. 1). By contrast, SI aerosols contained 5 times more water soluble organic nitrogen (WSON). Chemical speciation analyses of WSON revealed the presence of biogenic low molecular weight alkyl-amines, including mono-, di- and trimethylamine. Methylamines released by plankton incorporate into aerosols through reaction with sulphuric or methanesulphonic acids^5^, which are formed via oxidation of dimethylsulphide (DMS), another volatile of planktonic origin^6^. In fact, both methanesulphonic acid (MSA) and non-sea-salt sulphate (nss-SO_4_) were also enhanced in the ice-influenced air masses (Fig. 1).

*In situ* high time resolution monitoring of aerosol composition by High Resolution Time of Flight Aerosol Mass Spectrometry (HR-ToF-AMS) also revealed the ubiquitous occurrence of organic nitrogen (ON) compounds and their enhancement under sea ice influence (Fig. 1). To our knowledge, these are the first high time resolution aerosol composition measurements reported in the sea ice region of the Weddell Sea. As for the Atlantic Subantarctic region, a study conducted on Bird Island in the early summer reported aerosol compostion consisting of 47% sea salt, 21% nss-sulfate, 22% organics (including MSA and ON) and 8% ammonium^7^. These figures reflect a higher organic load than in our open-ocean aerosols, which average 85% sea salt, 5% nss-sulfate, 5% WSOC, 2% MSA, 2% ON and 1% ammonium (Fig. 1). In the previous study, however, the high content in organic nitrogen components - including amino acids and amines - was attributed to emissions from densely populated colonies of the island fauna^7^.

**Figure 2:**
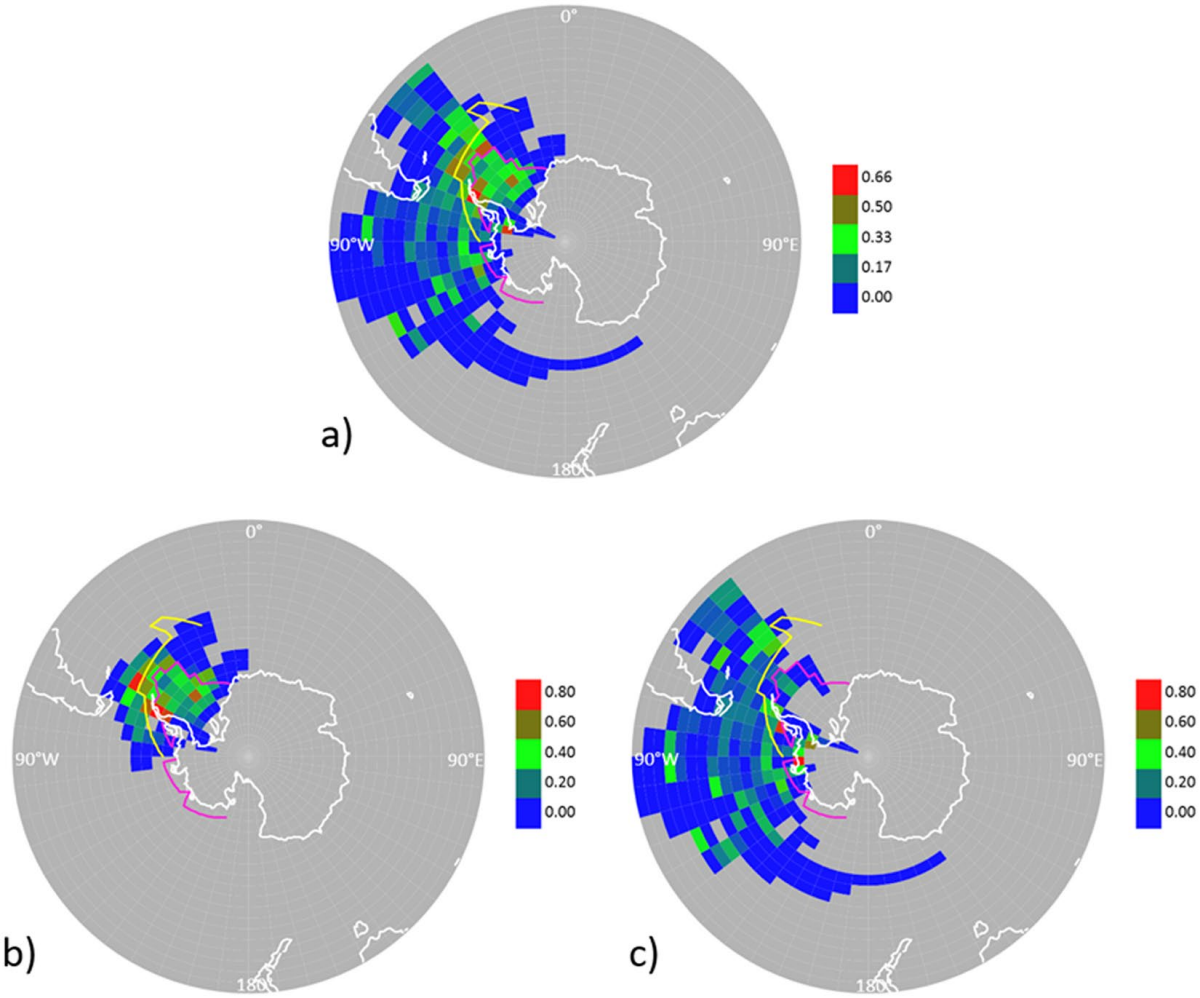
Apportioning of the origin of organic nitrogen in aerosols by Probability Source Contribution Function (PSCF) analysis. Continuous aerosol mass spectrometry data were combined with air-mass back trajectory analysis to quantify the probability of pixels to be the source of high ON concentrations in aerosols (defined as above the 3rd quartile). (**a**) Throughout the entire PEGASO cruise; (**b**) for the first half of the cruise, 8–21 January; (**c**) for the second half of the cruise, 22–31 January. Colour scale indicates PSCF weighting factors. The pink line indicates sea ice extent in January-February 2015. The yellow line indicates the approximate location of the Southern Boundary of the Antarctic Circumpolar Current (SBACC) within the 19–90° W sector, which defines the extent of major Weddell Sea ice influence on the contiguous ocean. This plot was created using the R software (R Development Core Team, R i386 3.3.2; www.r-project.org).

In our work, a geographical source apportionment for our high-resolution aerosol components was conducted by Probability Source Contribution Function analysis of air mass back trajectories. This analysis showed that the most probable, homogeneous source region for the measured aerosol ON was the ice-covered Weddell Sea and its marginal zone of ice-influenced ocean, along with some spots around the Antarctic Peninsula and the South Georgia phytoplankton bloom (Fig. 2).

Biogenic organic nitrogen and sulphur compounds have been shown to play synergistic roles in new particle formation^8^. We monitored the number of particles in the smallest size range (1–3 nm) in real time, and found an enhancement of almost 3 fold (189 cm^−3^ versus 69 cm^−3^) in SI air relative to OW air (Fig. 1). Although we cannot directly link any compound to the observed new particles, these data provide additional evidence of secondary aerosol production under sea ice influence.

**Figure 3:**
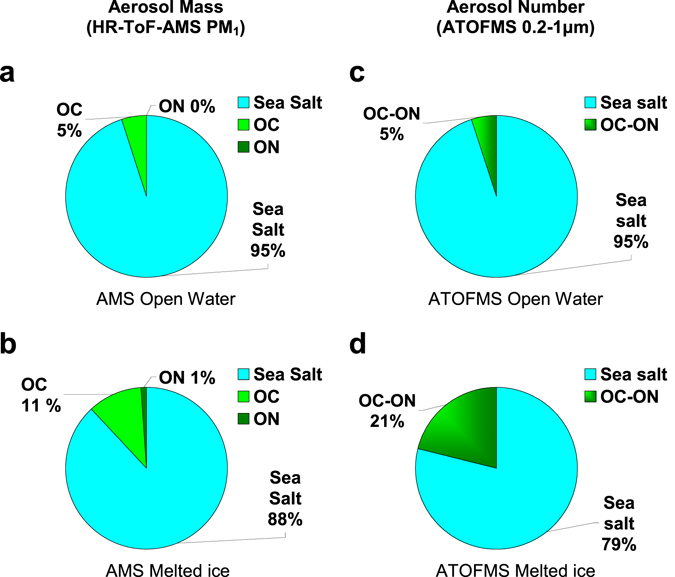
Single-particle composition of aerosols generated by a bubbling tank. (**a,c**) from bubbled open ocean water, and (**b,d**) from bubbled melted sea ice. (**a,b**) Contribution to the mass of aerosols < 1 µm; (**c,d**) proportion of aerosol numbers contributed by internal mixtures. OC: organic carbon; ON: organic nitrogen. Note that in **c** and **d**, OC-ON refer to internal mixtures of organic carbon and nitrogen with sea salt.

Single particle analysis of the ambient aerosols with on-line Aerosol Time-Of-Flight Mass Spectrometry (ATOFMS) further showed an 8-fold enhancement of ON in ice-influenced air masses. Moreover, of all ON-containing particles analysed, 11% were internally mixed with sea salt. Whilst this confirms a major secondary aerosol pathway for aerosol ON formation, it also shows an unneglectable contribution of sea-spray-associated ON. The surface ocean is rich in colloidal and particulate organic matter including proteinaceous gels^9^, bacteria and viruses, which can act as sources of primary N-containing aerosol by bursts of entrained air bubbles^10, 11^. Whether sea ice could also be a source of primary ON aerosol was unknown. To test this, we collected sea-ice samples from the northernmost margin of the Weddell Sea. Sample chunks were selected amongst ubiquitous, rafted sea ice floes discoloured by ice algae colonization at freeboard level. They were melted in a bubbling tank designed to produce sea spray. Mass spectrometric analyses of generated particles were compared with those produced by bubbling sea water. AMS characterisation showed that sea spray from melted ice contained more than twice as much organic carbon as seawater spray (11% vs 5%) and as much as 0.3–1% of ON, which was almost undetectable from seawater (Fig. 3). ATOFMS single-particle information on the mixing state revealed that 21% of the generated particles from sea ice were an internal mixture of sea salt with organic nitrogen and carbon, versus only 5% in seawater spray (Fig. 3). Measurements of black carbon and metals in the same sea-spray aerosols ruled out contamination artifacts.

**Figure 4:**
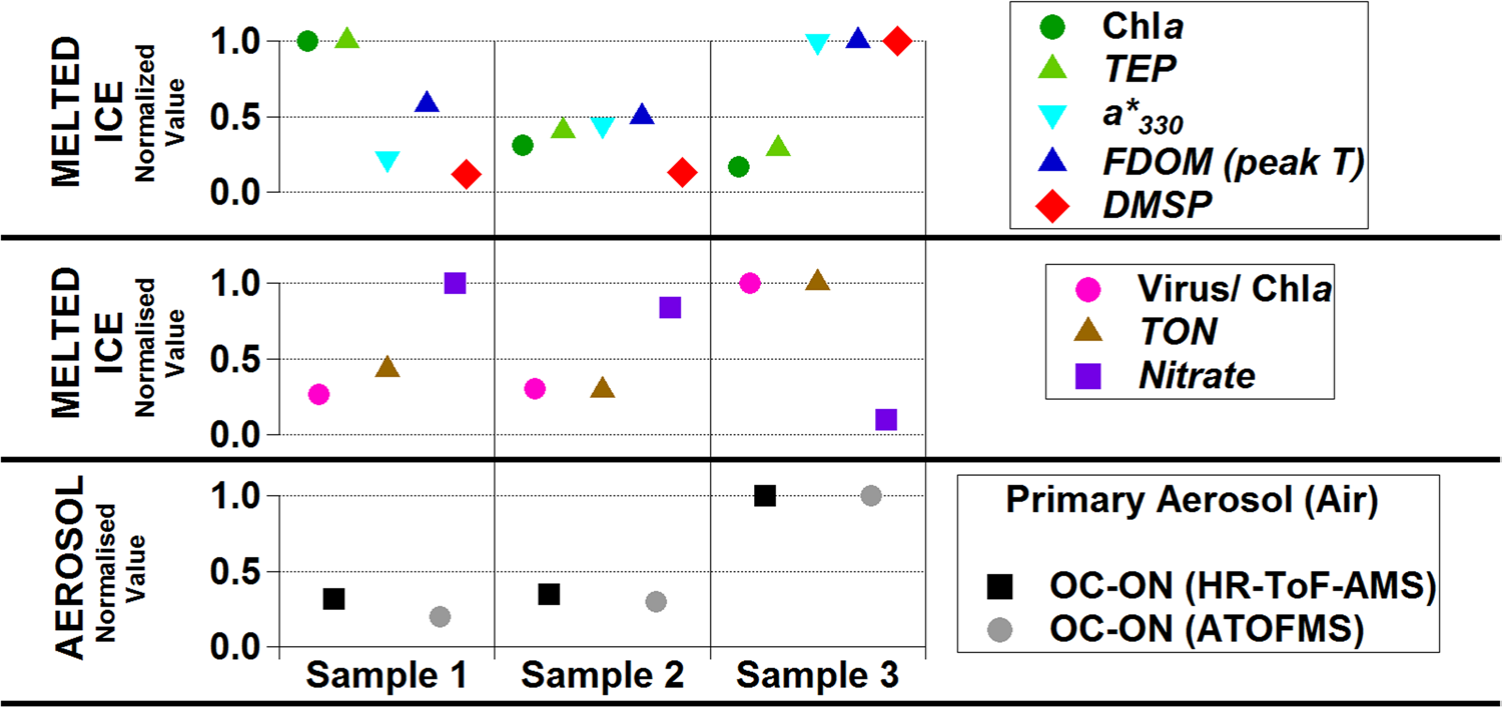
Concentrations in melted sea ice samples and the corresponding sprayed aerosols. Three ice samples were melted and analysed for a number of variables: Chl*a* is chlorophyll *a*; TEP is transparent exopolymer particles; a*_330_ is light absorption at 330 nm corrected for baseline absorption, corresponding to the concentration of mycosporine-like aminoacids (MAA); FDOM peak T is the fluorescence of organic matter at the excitation/emission wavelengths 280/350 nm; DMSP is dimethylsulphoniopropionate; TON is total (particulate + dissolved) organic nitrogen; OC-ON is mixed organic carbon and nitrogen; HR-ToF-AMS is high-resolution time-of-flight aerosol mass spectrometry; ATOFMS is aerosol time-of-flight mass spectrometry. For the clarity of comparisons, all values are normalised to the maximum.

These results indicate that sea ice contained ON that, upon release by ice melt, was susceptible to incorporation into aerosol. Indeed, sprayed aerosol ON content was proportional to the ON content of melted sea ice (Fig. 4). We then explored the origin of ON compounds in the sea ice. Brine channels in ice harbour dense microbial communities^1^. Chlorophyll *a* concentrations in the sea ice samples (8–48 µg L^−1^) were higher than those found in surrounding seawater (0.3–2 µg L^−1^) and within the range previously found in similar rafted sea ice^12^. Ice algae were largely dominated by diatoms of the genera *Fragillariopsis* and *Nitzschia*, commonly found in polar sea ice and surrounding waters^13^. Brine channels are also known to harbour abundant organic matter^1^ that remains largely uncharacterized. Consistent with previous reports^14, 15^, we measured concentrations of carbon-rich exopolymeric particulate matter in the ice which were ten times higher than in seawater samples. However, neither these C-rich particles nor chlorophyll *a* concentrations in sea ice samples were positively correlated to endogenous and sprayed ON (Fig. 4). This suggests that algal biomass and production in the sea ice brines is not a good predictor for aerosol-forming ON, probably because the physiological state of microorganisms plays an important role. Indeed, the parallel patterns of ON and the ratio of large viruses to chlorophyll *a*, and the opposite pattern to nitrate concentrations, suggest that the maturity and decay of the algal assemblage would enhance sea ice ON concentrations. The fluorescence and light absorbance signals for protein-like dissolved organic matter were positively correlated to sprayed ON (Fig. 4). Fluorescence has been previously used to suggest peptide enrichments in Antarctic sea ice^16^. Moreover, ice brine phytoplankton are known to produce copious amounts of mycosporine-like amino acids (MAA) as chemical sunscreens for protection from UV radiation^17^. MAA have a distinct light absorption pattern that allows relative quantification^18^. MAA in our ice samples were proportional to the ON contents in the sprayed aerosols (Fig. 4). Altogether, these results clearly depict sea ice as an organic nitrogen-rich matrix due to the embedded microbiota.

When sea ice breaks and melts, a water of lower salinity and higher buoyancy tends to accumulate at the ocean’s surface among the ice floes, and similar behaviour is expected for the released biogenic matter^1, 9^. It is plausible, therefore, that the non-volatile forms of organic nitrogen contained in ice floes, like proteins, MAA and nitrogen osmolytes, are readily susceptible to air-sea exchange by sea spray generation upon bubble bursting. Our knowledge of sea spray production in sea ice regions is very limited, except for open leads in the Arctic^19, 20^. Interaction of ocean waves with sea ice edge and floes, including splashes, whirls and other turbulent structures, as well as bubbles generated underneath the sea ice surface, may provide significant pathways for the transfer of biological material and sea salt from the surface polar water into the atmosphere^21^.

**Figure 5:**
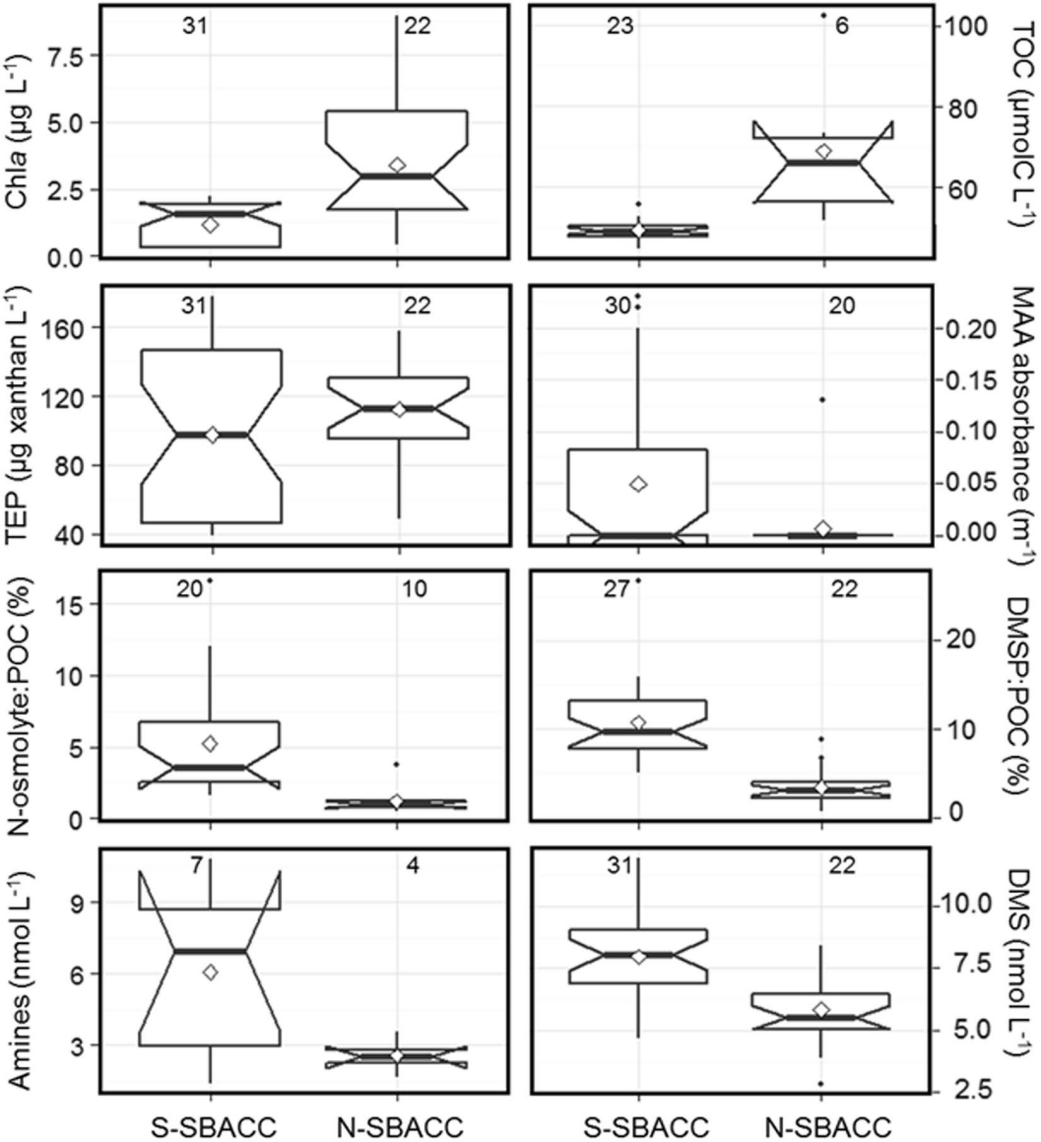
Seawater component occurrence south and north of the Southern Boundary of the Antarctic Circumpolar Current (S-SBACC and N-SBACC, respectively). The bold line at the centre is the median, the diamond is the mean, and the hinges represent the first and third quartiles. The lower end of the vertical bar is the minimum value, and the upper end is 1.5 times the inter-quartile range. The numbers inside each chart are the number of measurements. N-osmolytes (glycine betaine + choline) and DMSP are shown as their proportion to total particulate organic carbon. All median pairs are significantly different (*p* < 0.05) except for transparent exopolymeric particles (TEP) and MAA absorbance. In the case of MAA, the mean is more meaningful than the median, because the many zeros in both populations drive the medians to zero; means are significantly different.

Moreover, the most labile of the ice-released ON will be quickly degraded into volatile compounds, including alkyl-amines, by marine bacteria^22, 23^, thus favouring gaseous nitrogen emission fluxes from ice-pack openings and the marginal ice zone. This is similar to what occurs with sulphur: sea ice is an organic sulphur-rich environment because ice algae generally contain high intracellular levels of dimethylsulphoniopropionate (DMSP) for osmoregulation and cryoprotection^12, 24^. Release of DMSP from ice enhances the emission of its volatile breakdown product, DMS, through microbial action^6, 24^. Besides this degradation of organic material released from brine channels, sea ice promotes formation of nitrogen and sulphur volatiles in a more indirect way, by triggering oceanic phytoplankton blooms along the seasonally receding ice edge^1^. In the Weddell / Scotia Sea region of our study, the border of sea ice influence in ice-free waters was defined by the position of the Southern Boundary of the Antarctic Circumpolar Current (SBACC)^25^, around 60°S (Supplementary Figures 4 and 5). It is worth noting that, although waters south of the SBACC had lower chlorophyll *a* and organic carbon concentrations, and similar concentrations of exopolymers to those of the northern side, they had higher occurrence of MAA, higher proportions of the algal osmolytes glycine betaine, choline and DMSP, and higher concentrations of their degradation volatiles methylamines and DMS (Fig. 5). Bubbling tank experiments with ice-free waters showed that waters nearer the sea ice edge were more likely to supply ON in sprayed aerosols: according to ATOFMS analyses, 31% of total particles in the S-SBACC region contained ON, versus 15% in the N-SBACC region.

This study demonstrates that the microbiota throughout the sea ice region are an important contributor to aerosols by emission of aerosol-forming volatile and non-volatile ON, which adds to previously reported emissions of volatile sulphur^24^ and organic carbon microgels^19^. The relative importance of biogenic sources of primary versus secondary N in aerosols cannot be fully assessed at present, but our single-particle analyses of ambient aerosols discussed above (Fig. 3) indicates that, whilst secondary aerosols comprise the vast majority, primary ON aerosols should not be neglected. Amines are known to trigger formation of new aerosols^4, 8^ and allow their further growth into cloud-seeding particles. Whether primary ON-rich particles behave similarly remains unknown but, in the Arctic, biogenic C-rich primary aerosols from the sea ice region have already been identified as an efficient source of cloud condensation nuclei^19, 26^.

Past and on-going climatic changes are amplified in the polar regions^2^. As climate warms, energetic wave events are expected to occur more frequently^27^. Even though there is still much unknown complexity in the atmosphere–ocean–sea-ice system to enable accurate projections of Antarctic sea ice extent^28, 29^, increased storm frequency and reduced sea ice thickness will facilitate the breakup of sea ice by waves^30^ and strengthen sea ice-air interactions. Our results show that the sea ice region is a previously overlooked^31^ source of organic nitrogen in climate-relevant aerosols. This study calls for better representation of the sea ice-ocean-atmosphere biogeochemistry in Earth system models.

## Methods

### The cruise

The PEGASO (Plankton-derived Emissions of trace Gases and Aerosols in the Southern Ocean) cruise was conducted on board de RV Hesperides in the regions of Antarctic Peninsula, South Orkney and South Georgia Islands from 2 January to 11 February 2015.

### Aerosol counts and sizing

Particles in the range 1–3 nm were measured every 2 minutes with a Particle Size Magnifier (PSM, consisting of a Airmodus A10 operated with a TSI3775 condensation particle counter). Particle size distributions in the 10–500 nm size range were measured with a Scanning Mobility Particle Sizer (SMPS; DMA TSI 3080 and CPC TSI 3025) at 5 minute resolution.

### Aerosol sampling on filters

Ambient aerosol was sampled by means of a multistage impactor and a high volume sampler in parallel, from the ship upper deck. The Berner impactor (type LPI80, Hauke), mounting Tedlar foils as sampling substrates, collected particles on five stages with 50% particle upper cut off points at 0.14, 0.42, 1.2, 3.5 and 10 μm aerodynamic diameter (Dp), at a flow rate of 80 LPM. The high volume sampler (TECORA ECO-HIVOL, equipped with Digitel PM1 sampling inlet) collected ambient aerosol particles with Dp < 1 μm on pre-washed and pre-baked quartz-fibre filters, at a controlled flow of 500 LPM. Sampling was allowed only when the samplers were upwind the ship exhaust with a relative wind speed threshold of 5 m s-1. Due to the necessity of collecting sufficient amounts of samples for detailed chemical analyses, sampling time was of the order of ~50 h for each sample. Samples were stored at −20 °C until the chemical analyses.

### Chemical analyses of air filters

Aerosol samples were extracted in MilliQ water by sonication (30 min). Extracts were analysed by ion chromatography for the quantification of water soluble inorganic ions, organic acids (acetate, formate, methanesulphonate, oxalate)^32^ and low molecular weight alkyl-amines (methyl-, ethyl-, dimethyl-, diethyl- and trimethylamine)^5^. An IonPac CS16 3 × 250 mm Dionex separation column with gradient MSA elution and an IonPac AS11 2 × 250 mm Dionex separation column with gradient KOH elution were deployed for cations and anions, respectively. The water soluble organic carbon (WSOC) and water soluble total nitrogen (WSTN) content of the samples were quantified using a TOC-TN thermal combustion analyser (Multi N/C 2100 by Analytik Jena)^33^. The water soluble organic nitrogen (WSON) was calculated as the difference between the WSTN and the inorganic nitrogen deriving from ammonium, nitrate and nitrite ions, as detailed elsewhere^5, 34^. All the chemical species, with the exception of WSOC, were determined from the Berner impactor samples. WSOC concentration was determined from the high volume samples in order to improve accuracy. WSOC measurements are less sensitive than Ion Chromatography ones, therefore dividing the sample over the different stages of the impactor caused an enhancement of the measurement uncertainty, because of the presence of stages with WSOC concentration below or very close to the detection limit. This is not the case with the high volume sampler, which collects all the submicron aerosol mass in only one filter. Comparison of the results for ionic species measured on both substrates, demonstrate consistency between the two samplers.

### Real time mass spectrometry

The size resolved non-refractory chemical composition of submicron aerosol particles was measured with an Aerodyne High Resolution Time of Flight Aerosol Mass Spectrometer (HR-ToF AMS, Aerodyne, Billerica, MA). The instrument was described by DeCarlo *et al*.^35^. HR-ToF AMS was routinely calibrated according to the methods described by Jimenez *et al*.^36^. ON AMS is defined as total ON containing m/z fragments detected online by HR-ToF-AMS. The aerosol time-of-flight mass spectrometer (ATOFMS, TSI model 3800) collects bipolar mass spectra of individual aerosol particles. Ambient aerosol is focused into a narrow particle beam for sizes between 0.1 and 1.5 μm.

### Black carbon and metals

Black Carbon was measured by Single Particle Soot Photometer^37^ (SP-2) and metals by ATOFMS^38^.

### Air mass trajectories

Five-day back trajectories arriving at the ship’s position at 00:00, 06:00, 12:00 and 18:00 every day were calculated using the BADC Trajectory Service. In total, 117 air mass back trajectories were obtained. Following the Bergeron classification, two main air masses described the study area: continental Antarctic - cAA - (air masses that are extremely cold and dry due to their continental source region between 60°S and 90°S) and Maritime Polar - mP - (from open water regions). Simple calculations were made to assign each 5-days trajectory to the types of surface cover overflown. The Polar Stereographic map classified each of 1024 × 1024 24 km grid cells as land, sea, ice and, from this information, the percentage of time each trajectory spent over each surface type, and particularly over sea ice, could be calculated. A similar calculation was repeated but using daily maps of sea ice percentage concentration measured on a 12.5 km grid. The percentages assigned to each trajectory step allow a ‘spectrum’ of sea ice concentration from 0 to 100% (5% width) to be calculated, as shown in Supplementary Figure 1. Sea ice concentration was derived from satellite microwave data^39^ available at IFREMER. This same analysis allowed assigning air mass trajectories to the aerosol samples collected on filters. Of a total of 8 filter samples, three (PE24–28–06) had spent most of the time (79%) over open water, and three (PE09–13–18) had spent 73% of the time over sea ice and the sea ice marginal zone (Supplementary Table 1).

### Probability Source Contribution Function analysis

To identify the potential source areas of the observed ON-rich aerosol, potential source contribution function (PSCF) analysis was applied^40^. The procedure follows closely the one described by Chang *et al*.^41^. In the present work, five-day back trajectories (HYSPLIT4 with GDAS data)^42^ were calculated four times a day during the cruise period, with arrival position 100 m above the ship. Organic nitrogen AMS data, filtered for black carbon (BC) < 1 ng m^−3^ to avoid contaminations from the ship, were averaged over 6 hours periods (from 3 hours before the arrival time of each back trajectory to 3 hours afterwards). The southern hemisphere was divided into 2.5° latitude x 7.5° longitude grid cells. The conditional probability that the air passing through the ij-th cell had a high aerosol concentration when arriving to the ship position is given by the ratio nij/mij, where nij is the number of trajectories with segment endpoints in cell ij, and mij is the subclass (mij < nij) of trajectories connected to organic nitrogen concentrations above a threshold defining “high concentrations” (in this case, the 3d quartile of the ON database).

Weighting factors were applied to the grid cells, according to the following scheme:

$$\begin{array}{rcl}{\rm{W}} & = & 1.0\,{\rm{when\; nij}} > {\rm{N}}2\\  & = & 0.8\,{\rm{when\; N}}1 < {\rm{nij}} < {\rm{N}}2\\  & = & 0\,{\rm{when\; nij}} < {\rm{N}}1.\end{array}$$

where nij is the number of trajectories passing for each cell in the study period and N1 = 60 * cos(latitude), and N2 = 300 * cos(latitude). The cosine factor is used to account for the changing grid cell size with varying latitude.

### Aerosol generation tank experiments

An airtight high grade stainless steel tank (200 L) was half filled with a constant flow of seawater pumped from a depth of 4 m by the flow-through pumping system of the ship. Water was dropped from the top of the tank as a plunging jet at a flow rate of 20 L min^−1^. The entrained air formed bubbles that, upon bursting, produced sea spray aerosol, as reported in O’Dowd *et al*.^43^. Particle-free compressed air was blown into the tank headspace (120 ml min^−1^), which had outlet ports leading to an Aerosol Chemical Speciation Monitor (ACSM) and a Scanning Mobility Particle Sizer (SMPS) for aerosol size distributions (10–500 nm). In parallel, bubble-bursting aerosol generation experiments with the same samples were performed using a square glass tank (10 L) that was filled with about 3 L of either seawater or melted sea ice, sealed with a stainless steel lid and continuously flushed with particle-free air (11 L min^−1^) as described elsewhere^44^. Sprayed aerosol size and composition was monitored with a HR-ToF-AMS and an ATOFMS.

### Hydrographic measurements and seawater sampling

Seawater salinity and temperature were recorded continuously via the flow-through thermosalinograph SBE 21 SeaCAT. The water current velocity was measured with the Shipboard Acoustic Doppler Current Profiler (SADCP) “Ocean Surveyor” at 75 khz. Seawater samples were collected from a depth of 4 m using either the uppermost Niskin bottle of the CTD rosette or the ship’s flow-through underway pumping system.

### Allocation of main surface ocean currents and fronts

Positions of the main hydrographic fronts and surface ocean water masses were determined on the basis of the seminal scheme of Orsi *et al*.^25^. Gradients in the continuous record of seawater, salinity (Supplementary Figure 3) and surface current direction and velocity along the cruise track were combined with synoptic modeling data obtained from the Global Real-Time Ocean Forecast System (Global RTOFS) to allocate the critical hydrographic boundaries (Extended Data Figure 4).

### Sea Ice samples

Three sea ice samples were collected on three different days at the northern edge of the Weddell Sea marginal ice zone, south west of South Orkney Islands. From a zodiac boat, ice chunks were selected for their brownish colour indicating colonisation by algae. Once in the on board laboratory (within one hour of collection), about 0.1 m^3^ of sea ice was introduced into the aerosol generation tank and was melted in the dark for 24 hours. Initial melt was facilitated by adding about 1/5 volume of sea water collected at the ship location near sea ice. Ice melt water was recirculated into plunging jet to generate bubbles and aerosols.

### Phytoplankton biomass and composition

Surface ocean and melted ice chlorophyll *a* concentrations were determined by filtration on glass fibre filters followed by extraction with 90% acetone at 4 °C in the dark for 24 hours. Fluorescence of extracts was measured with a calibrated Turner Designs fluorometer. No phaeophytin corrections were applied. Samples were also taken and fixed with formalin-hexamine 1% for quantitative phytoplankton determination by the inverted microscope method^45^.

### Viral abundances

Samples (2 ml) were fixed with glutaraldehyde (0.5% final concentration), refrigerated, quick frozen in liquid nitrogen and stored at −80 °C. In the lab, they were stained with SYBR Green I and counted with FACSCalibur flow cytometer with a blue laser (488 nm). Only high-fluorescence viral populations, considered to infect eukaryotes, were used in this study^46^.

### Sea-ice total organic nitrogen

Aliquots of melted sea ice were frozen at −20 °C until analysis of nitrate, nitrite and ammonium with an automated segmented flow analyser. Organic nitrogen was determined upon digestion (120 °C for 30 min) with alkaline persulfate and subtraction of inorganic nitrogen concentrations^47^.

### Chromophoric (CDOM) and fluorescent (FDOM) organic matter

CDOM absorption spectra were measured by spectrophotometry on board, and the peak intensity of absorption at 330 nm was extracted from the baseline absorption as a measure of mycosporine-like aminoacid (MAA) concentration. FDOM excitation/emission matrices were also analysed on board using a Perkin Elmer LS55 luminescence spectrometer equipped with a xenon discharge lamp equivalent to 20 kW for a duration of 8-µs. Excitation at 280 nm and emission at 350 nm (so-called peak T) was taken as indicator of protein-like substances^48^.

### Transparent exopolymer particles (TEP)

TEP were analysed following the colorimetric method^49^. Samples (160–450 mL) were filtered through 25 mm diameter 0.4 µm pore size polycarbonate filters (DHI). The filters were stained with 500 µL of pre-calibrated (with a xanthan gum solution) Alcian Blue (0.02%, pH 2.5) for 5 s and rinsed with MilliQ water. They were soaked in 80% sulphuric acid for 3 h and the absorbance of the extract was determined at 787 nm in a Varian Cary spectrophotometer. Duplicates were taken for each sample. Results were corrected for blanks consisting of unused filters stained with Alcian blue.

### Total (TOC) and particulate (POC) organic carbon concentrations

For TOC, 30 mL samples of sea water or melted sea ice were collected in acid-cleaned polycarbonate bottles, and stored in the dark at −20 °C until analysis. They were analysed in triplicate with a Shimadzu TOC-LCSV, with MilliQ water as a blank, potassium hydrogen phthalate as the calibration standard, and deep Sargasso Sea water as the reference. For POC, 250–2000 mL of seawater were filtered through pre-combusted (450 °C, 4 h) 25 mm glass fiber filters, frozen at −20 °C and analysed with a Perkin Elmer 2400 CHN analyzer.

### Aqueous MA and DMS concentrations

To determine methyl-amine concentrations, 850 mL seawater samples were gravity filtered through 47 mm GF/F filters into 1 L high density polyethylene (HDPE) bottles containing HCl (10 mL, 11.6 M), and stored at 4 °C until analysis. The seawater was saturated with NaCl and adjusted to pH 13.4 with sodium hydroxide. Methylamines were extracted from the water sample by solid phase microextraction approach^50^. A polydimethylsiloxane/divinylbenzene fibre was exposed in the headspace above the water sample, and the extraction performed for 2.5 h at 60 °C. The methylamines were then resolved and detected using a GC with NP detector. Calibrations were performed using external matrix-matched standard solutions containing mono-, di- and trimethylamine in the range 0.13–13.3 nM. DMS was determined, along with other volatiles, with a purge and trap GC with MS detector. Samples of 25 ml of seawater pre-screened through 200 µm were filtered on-line through GF-F, injected and sparged with He for 12 min. Calibration was performed with DMS solutions generated by dissolution and hydrolysis of solid DMSP in high purity water.

### Phytoplankton DMSP, glycine betaine and choline content

For DMSPt (particulate + dissolved, largely particulate) analysis, two pellets of NaOH were added to 30 mL of sample for hydrolysis to DMS, for at least 24 h at room temperature in the dark. Aliquots of 0.1 to 1 mL were injected into a purge flask with high purity water, sparged for 4–6 min. and analysed for evolved DMS with a purge and trap GC system with FP detector^51^. DMSPt concentrations were calculated by subtraction of the endogenous DMS. Calibration as for DMS. For glycine betaine and choline, 10 mL of sample was gravity filtered onto 47 mm GF/Fs. Filters were snap frozen and stored at −80 °C until analysis. Glycine betaine and choline were extracted from filters into 15 mL 12:5:1 methanol:chloroform:water containing internal standard (d_11_-glycine betaine) by soaking (1 hr). Solutions were clarified by centrifugation and analysed by LC/MS^52^. To calculate the proportion of POC contributed by DMSP or the N-osmolytes, their concentration in nmol L^−1^ was multiplied by 5 to account for 5 C atoms per molecule, and divided by 1000 and by the POC concentration in µmolC L^−1^.

## Electronic supplementary material

Supplementary Information
