# Supplementary material for: Antarctic sea ice region as a source of biogenic organic nitrogen in aerosols
Source: Sci Rep. 2017 Jul 20;7:6047. doi: 10.1038/s41598-017-06188-x (PMC5519629; doi:10.1038/s41598-017-06188-x)
Supplement: Supplementary file 1 — Supplementary Information [file 41598_2017_6188_MOESM1_ESM.pdf]

SUPPLEMENTARY INFORMATION

Antarctic sea ice region as a source of biogenic organic nitrogen in aerosols

Manuel Dall'Osto, Jurgita Ovadnevaite, Marco Paglione, David C. S. Beddows, Darius Ceburnis, Charlotte Cree, Pau Cortés, Marina Zamanillo, Sdena O. Nunes, Gonzalo L. Pérez, Eva Ortega-Retuerta, Mikhail Emelianov, Dolors Vaqué, Cèlia Marrasé, Marta Estrada, Montserrat Sala, Montserrat Vidal, Mark F. Fitzsimons, Rachael Beale, Ruth Airs, Matteo Rinaldi, Stefano Decesari, Maria Cristina Facchini, Roy M. Harrison, Colin O'Dowd, Rafel Simó

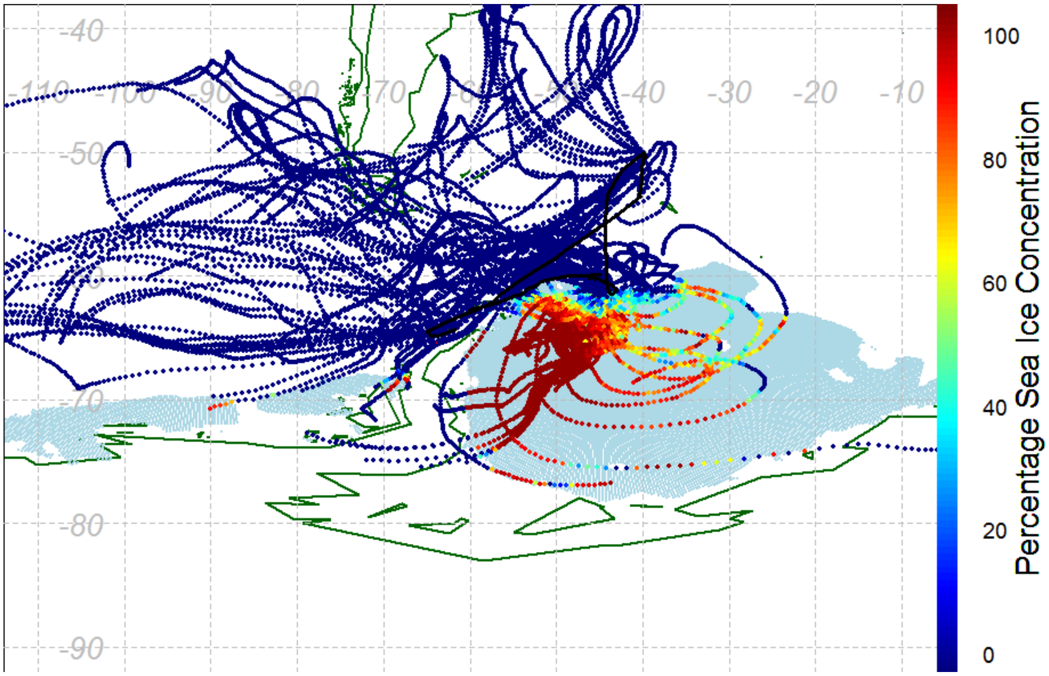

Supplementary Figure 1: Air mass trajectory analysis and source region. The black solid line is the cruise track. The colours on the air trajectories indicate the percentage of time spent overflying sea ice (see colour bar). The 117 air mass back trajectories are classified according to two main source regions: Open Water (OW, mainly blue for the lowest percent of sea ice) and Sea Ice (SI, in green-red for high percent of sea ice). The back trajectories were calculated backward for 5 days (120 hours). This plot was created using the R software (R Development Core Team, R i386 3.3.2; www.r-project.org).

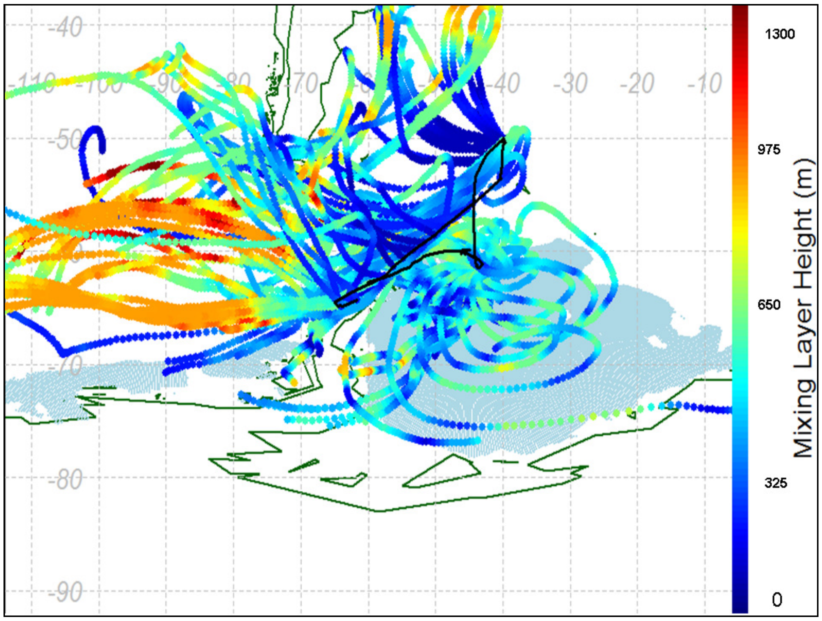

Supplementary Figure 2: Hourly Mixing Layer Heights (MLH, also referred as Mixing Depth MD) of the 5-days trajectories of the sampled air masses. Averages ± stdev were 463±57 m for the sea ice region and 537±151 m for the open ocean region. This plot was created using the R software (R Development Core Team, R i386 3.3.2; www.r-project.org).

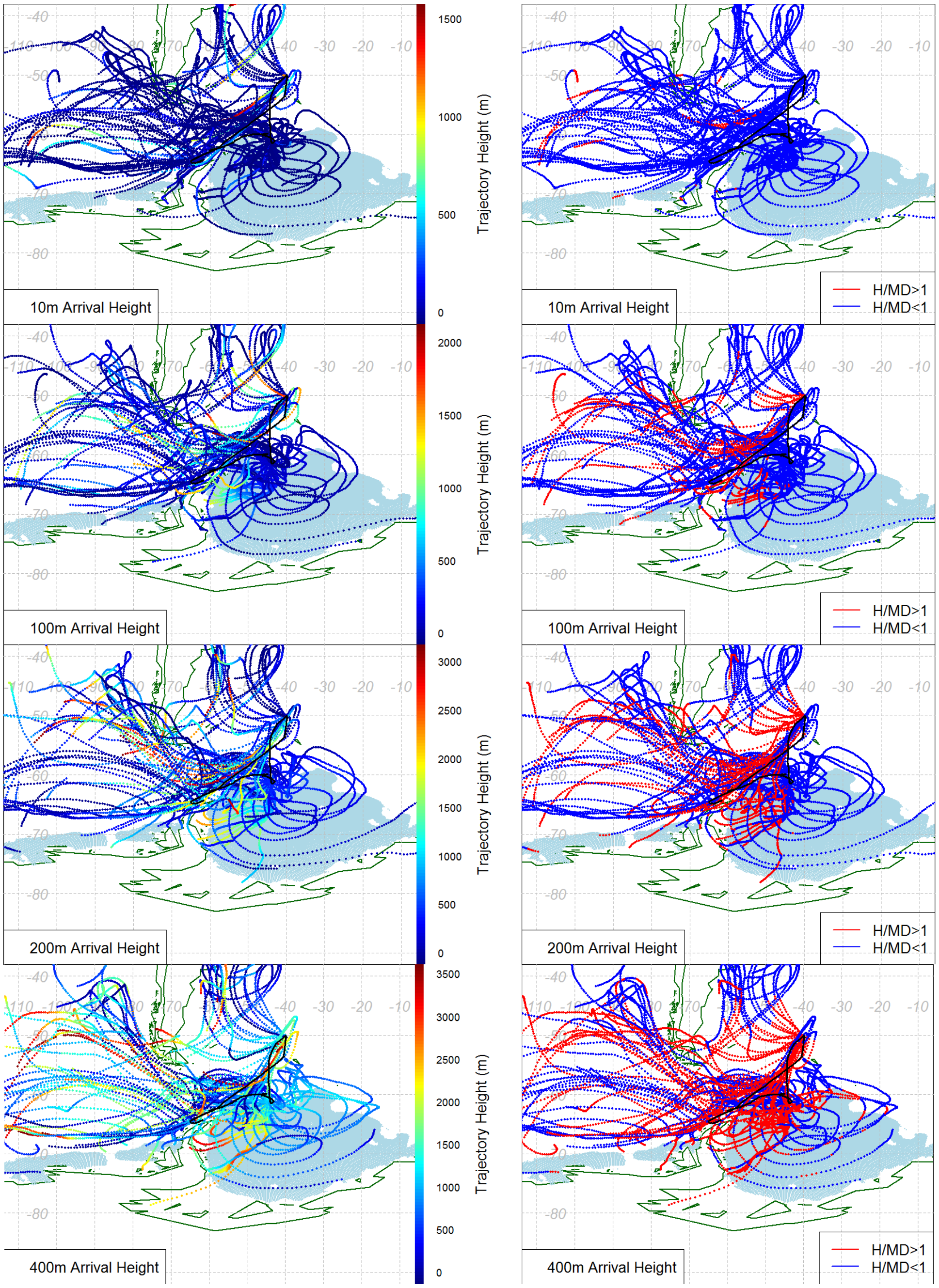

Supplementary Figure 3: Trajectory heights of the air mass arriving to four altitudes (10, 100, 200, 400 m) of the sampled mixing depth (MD, right). Portions of the air mass trajectories that were within the local MD (blue) or above the MD (red). H stands for trajectory height; MD stands for mixing depth (right plots). Note from Figure S2 that the MD was 450-550 m most of the time. It was found that - out of the 14,040 hours of computed air transport - the air masses ending up in the sampled MD had spent the majority of their time on the previous 5 days within the MD (see also Table S2 below). Only the air that ended up at 400 m (i.e., close to the MD top) on the sampling day, had a significant passage above the MD. Most importantly, there were no strong differences in mixing with the FT between the two

regions. When examining the back-trajectories travelling to the top of the MD (Figure S3, 400 m arrival height panels), air mass descent was occasionally observed from continental Antarctica, consistently with the quasi-permanent anticyclonic circulation forced by orography, and flowing from inner ice caps and the overlaying free troposphere, hence from regions where the sources of aerosol organic compounds are very scarce. A little more often during the campaign, under open ocean conditions, entrainment of free tropospheric air in the upper part of the MD was observed in conjunction with cyclogenesis in the roaring forties zone. However, being the general circulation at these latitudes from the west, most of these back-trajectories reached the research ship after crossing the Drake Passage and originated from vast regions in the south Pacific where it is hard to identify aerosol sources which are not the same marine boundary layer sources already accounted for in Figure 2 of the manuscript. Only when the research ship reached the north tip of the cruise around S. Georgia Islands, there is evidence of transport from the Argentinean coast area but very rarely from over the South American continent itself. This plot was created using the R software (R Development Core Team, R i386 3.3.2; www.r-project.org).

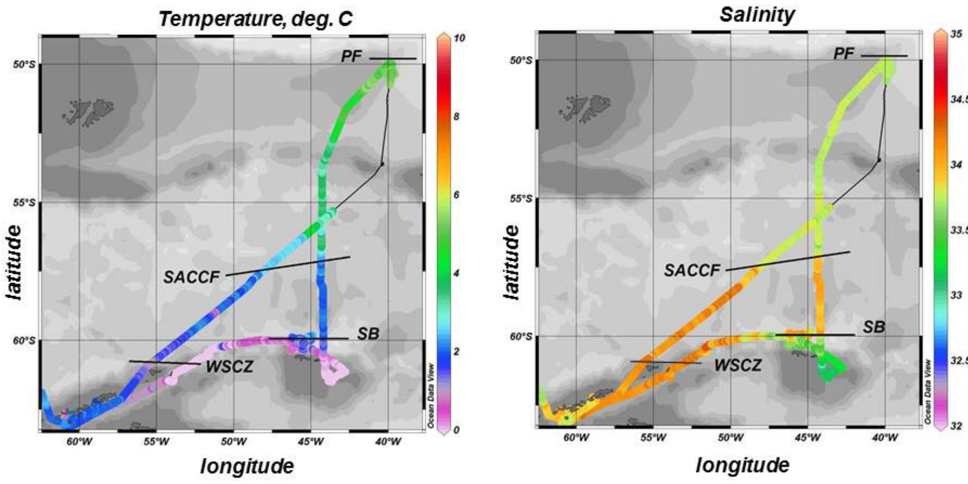

Supplementary Figure 4: Sea surface temperature and salinity and the position of the main oceanic fronts. Temperature and salinity recorded by the flow-through thermosalinograph were combined with surface current directions and velocities to allocate the main hydrographic fronts along the cruise track: Antarctic Polar Front (PF; 50ºS), Southern Antarctic Circumpolar Current Front (SACCF¸56.8ºS-57.2ºS), Southern Boundary of the Antarctic Circumpolar Current (SB; 59.9ºS), and Weddell Scotia Confluence Zone (WSCZ; 60.0ºS-60.8ºS). This plot was created using the R software (R Development Core Team, R i386 3.3.2; www.r-project.org).

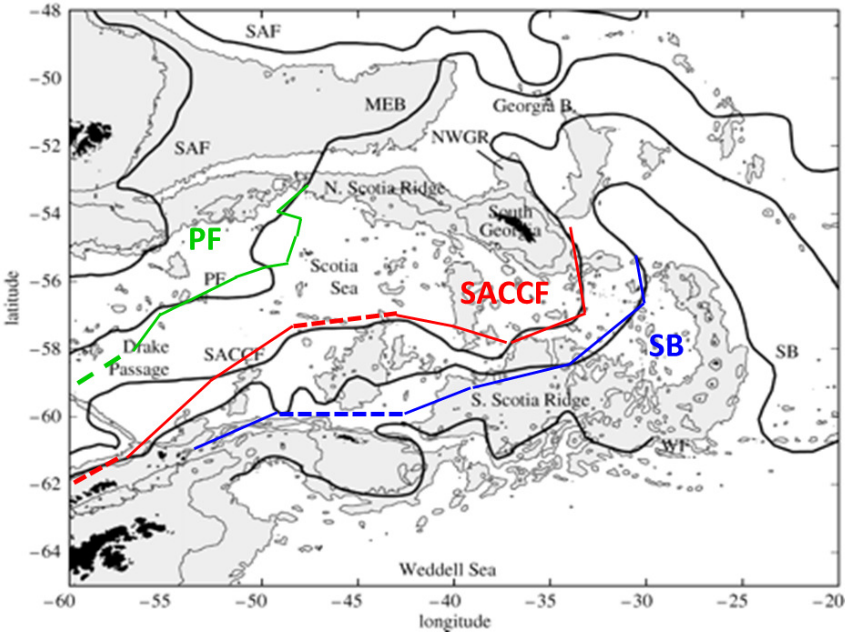

Supplementary Figure 5: Approximate synoptic locations of the main hydrographic fronts. Combination of the aforementioned measurements with synoptic modelling (see Methods) allowed drafting the hydrographic fronts, here superimposed to the seminal description by Orsi *et al*.[25] This plot was created using the R software (R Development Core Team, R i386 3.3.2; www.r-project.org).

Supplementary Table 1. Average percentage of air mass trajectory time (out of 120 h) spent over different areas (land, open water, sea ice, and inner open waters within the sea ice marginal zone) before being sampled for aerosols. Filter samples PE24, PE28 and PE06 were assigned to OW region; filter samples PE09, PE13 and PE18 were assigned to SI region. Note that percentages add up to 100%.

Region	***% Land***	***% Open water***	***% Total Sea ice***	***% Sea ice (packed)***	***% sea ice (inner open waters within the marginal ice zone)***
**OW region**	3	79	18	(0	18)
**SI region**	2	25	73	(48	25)

Supplementary Table 2. Percentages of time the air mass spent within the BL on the 5 days prior to sampling. This is computed for four different heights (10, 100, 200, 400m) within the sampled BL.

Sampling Region	10m	100m	200m	400m
**sea ice**	99%	92%	88%	66%
**open ocean**	98%	83%	77%	62%
